# The emergence of an imported variant of dengue virus serotype 2 in the Jazan region, southwestern Saudi Arabia

**DOI:** 10.1186/s40794-023-00188-8

**Published:** 2023-03-15

**Authors:** Ommer Dafalla, Ahmed A. Abdulhaq, Hatim Almutairi, Elsiddig Noureldin, Jaber Ghzwani, Omar Mashi, Khalid J. Shrwani, Yahya Hobani, Ohood Sufyani, Reem Ayed, Abdullah Alamri, Hesham M. Al-Mekhlafi, Zaki M. Eisa

**Affiliations:** 1 Saudi Public Health Authority, Jazan, Kingdom of Saudi Arabia; 2 Jazan University, Jazan, Kingdom of Saudi Arabia

**Keywords:** Dengue virus, Virus evolution, Infectious diseases, Imported variant, Serotype, Jazan, Saudi Arabia

## Abstract

**Background:**

Dengue virus (DENV) infection is a global economic and public health concern, particularly in tropical and subtropical countries where it is endemic. Saudi Arabia has seen an increase in DENV infections, especially in the western and southwestern regions. This study aims to investigate the genetic variants of DENV-2 that were circulating during a serious outbreak in Jazan region in 2019.

**Methods:**

A total of 482 serum samples collected during 2019 from Jazan region were tested with reverse transcription-polymerase chain reaction (RT-PCR) to detect and classify DENV; positive samples underwent sequencing and bioinformatics analyses.

**Results:**

Out of 294 positive samples, type-specific RT-PCR identified 58.8% as DENV-2 but could not identify 41.2%. Based on sequencing and bioinformatics analyses, the samples tested PCR positive in the first round but PCR negative in the second round were found to be imported genetic variant of DENV-2. The identified DENV-2 imported variant showed similarities to DENV-2 sequences reported in Malaysia, Singapore, Korea and China. The results revealed the imported genetic variant of DENV-2 was circulating in Jazan region that was highly prevalent and it was likely a major factor in this outbreak.

**Conclusions:**

The emergence of imported DENV variants is a serious challenge for the dengue fever surveillance and control programmes in endemic areas. Therefore, further investigations and continuous surveillance of existing and new viral strains in the region are warranted.

## Background

Dengue fever (DF), a mosquito-borne viral infection caused by the dengue virus (DENV), is a serious disease and economic burden in approximately 140 tropical and subtropical countries [1, 2]. An estimated 400 million DENV infections occur every year, resulting in 25,000 deaths, with 70% of cases in Asia [3, 4]. Furthermore, 3.83 billion people (approximately half of the global population) live in areas suitable for the virus [2]. DENV is transmitted by the bite of female *Aedes* mosquitoes, mainly *Aedes aegypti* and, to a lesser extent, *Aedes albopictus* [5]. Infection by DENV causes DF, a flu-like illness characterised by constant high fever, intense headache, retro-orbital pain, myalgia, joint pain, vomiting and rash, which occasionally develops into life-threatening dengue haemorrhagic fever (DHF) [6]. Dengue virus infection is diagnosed clinically and confirmed by detecting anti-dengue immunoglobulin G (IgG) and/or IgM antibodies, and non-structural 1 (NS1) antigen using serology or viral RNA using reverse transcription-polymerase chain reaction (RT-PCR) [7].

Dengue virus is a single positive-stranded RNA virus belonging to the *Flavivirus* genus and *Flaviviridae* family. It contains 10,700 nucleotides, with an 11-kb genome encoding three structural proteins: core (C), membrane (M) and envelope (E) proteins, and seven non-structural proteins: NS1, NS2A, NS2B, NS3, NS4A, NS4B and NS5 [8]. Genetic and antigenic characteristics show DENV is four distinct but closely related serotypes (DENV-1, DENV-2, DENV-3 and DENV-4), with an inter-serotype nucleotide variability of approximately 30% [9]. Each serotype is sub-divided into genotypes and lineages based on nucleotide variations of 6–8% and amino acid of 3% [10, 11]. Different DENV serotypes and genotypes demonstrate different levels of virulence and epidemic capacity [12, 13]. Thus, continuous phylogenetic evaluation of the circulating DENV genotype is crucial for monitoring outbreaks in endemic countries [13].

Saudi Arabia was considered a dengue-free country until the mid-1990s [14]. However, since 1994 there have been several outbreaks, with an increasing number in recent years; 2013 had the highest number of cases at 6,512, and approximately 3,000 were reported in 2019 [15–17]. Most cases were in Jeddah, followed by Jazan and Makkah regions [17]. Previous studies have reported all DENV serotypes in Jeddah, Makkah, Al-Madinah, Aseer and Jazan regions, with DENV-2 predominant followed by DENV-1 [18–20]. Since 2005, Jazan region of southwestern Saudi Arabia has been affected by many DENV outbreaks [15, 21]. A dramatic rise in cases of DENV infection in Jazan region occurred in 2019 and 2020 [22].

The Saudi Centre for Disease Prevention and Control (SCDC) in Jazan continuously monitors DENV infection, with laboratories utilising RT-PCR and following Lanciotti et al. [23] protocol. Previous studies evaluated the performance of some most commonly used conventional RT-PCR assays for detecting dengue viral RNA in clinical specimens and demonstrated that the widely used semi-nested Lanciotti protocol was the most sensitive method [24, 25]. During routine classification of DENV isolates at our laboratories, it was observed that a sizeable proportion of samples collected during the 2019 outbreak were first-round-PCR positive (DENV-specific PCR) but second-round-PCR negative (serotype-specific PCR). Recent studies from different countries have linked the emergence and co-circulation of variants of DENV, particularly DENV-2 and DENV-1, with the occurrence of large local outbreaks and the likelihood of dengue endemicity [26, 27]. Considering this, it was hypothesised that during the 2019 outbreak, apart from environmental, demographic, and host determinants, viral factors may have contributed to increased DENV spread in the region. Therefore, this study aims to investigate the genetic diversity of DENV-2 during the 2019 outbreak in Jazan region.

## Methods

### Study settings

Jazan region is in the southwestern part of the Kingdom of Saudi Arabia and stretches 300 km along the southern Red Sea coast (42.7076° E and 17.4751° N). Although it is the country's smallest region at 11,671km^2^, it has the highest population density, with 1.8 million people. Topographically, the region comprises of three zones: a highland zone (Fayfa Mountains) at an elevation of > 2500 m, a hill zone from 400–600 m and the Red Sea coastal plain below 400 m [28]. Jazan region contains many valleys, such as Wadi Baysh, and the Baysh dam, built in 2009, is one of the largest dams in the country. Jazan is the only region with indigenous malaria with few foci for malaria transmission and has had several outbreaks of DENV since 2005 [18, 29]. *Aedes aegypti*, the main vector for dengue fever, is prevalent in the region [30].

For this study, a total of 482 serum samples suspected of containing DENV were kindly supplied by the dengue fever control programme in Jazan. The samples were collected during the 2019 dengue fever outbreak. The ethical committee of the Saudi Centre for Disease Prevention and Control, Jazan, Saudi Arabia (SCDC) authorised the use of the samples (Ref. No. 23653 dated 23/10/2021). Patients were de-identified and study data was analyzed anonymously.

### RT-PCR

GeneJET RNA purification kits (Thermo Fisher Scientific, Waltham, MA, USA) were used to extract RNA following the manufacturer's instructions. The tests were performed using the DENV consensus primers (D1 forward primer 5-TCAATATGCTGAAACGCGCGAGAAACCG-3 and D2 reverse primer 5-TTGCACCAACAGTCAATGTCTTCAGGTTC-3) synthesised by Macrogen Company in Seoul, Korea, to amplify the 511 nucleotide fragment spanning capsid-pre-membrane (C-prM) junction of DENV serotypes 1–4 [23]. RT-PCR reactions were performed using RT-PCR system protocols (Promega, USA), a total volume of 50 μl following the manufacturer's recommended procedure. A thermal cycler (Eppendorf Corporate, Hamburg, Germany) was programmed as follows: RT step for 1 h at 42 °C to convert RNA to cDNA, initial denaturation for three minutes at 94 °C followed by 35 cycles of denaturation at 94 °C for 30 s, primers annealing at 55 °C for 1 min, primer extension at 72 °C for 2 min and a final extension for 5 min.

Nested PCR was performed in 50 μl reagent containing 25 μl GoTag®G2 green master mix from Promega, 5 μl of diluted (1:100) RT-PCR product, 50 pmol (final concentration 1 μM) of each forward primer D1 and serotype-specific reverse primers [TS1 (482 bp): 5’-CGTCTCAGTGATCCGGGGG-3', TS2 (119 bp): 5’-CGCCACAAGGGCCATGAACAG-3', TS3 (290 bp): 5’-TAACATCATCATGAGACAGAGC-3', and TS4 (392 bp): 5’-CTCTGTTGTCTTAAACAAGAGA-3'] [23]. The samples were subjected to initial denaturation at 94 °C for 3 min, 30 further cycles of denaturation at 94 °C for 30 s, primer annealing at 55 °C for 30 s, elongation at 72 °C for one minute and final extension for five minutes. In each cycle, negative and positive controls were included. The RT-PCR and nested products were run in 1.5% agarose gel electrophoresis and stained with ethidium bromide. A Gel Doc XR imaging system (Bio-Rad, USA) was used for visualisation.

### Sequencing and bioinformatics analysis

Partial sequencing was performed by Macrogen Company (Seoul, Korea) for RT-PCR products using D1 primer. Sequence obtained have a length of 449 nucleotides and correspond to positions 181–629 of the C-prM region. Then, sequences were examined for similarity using the basic local alignment search tool (BLAST) (https://doi.org/10.1016/s0022-2836(05)80360-2) and compared to DENV serotype reference sequences (accession numbers: NC_001477.1, NC_001474.2, NC_001475.2, and NC_002640.1). The sequenced sample alignment was performed using version 7.508 of MAFFT software (https://doi.org/10.1093%2Fmolbev%2Fmst010). The pairwise alignment of coding regions for nucleotides were analyzed using BioEdit sequence alignment editor (v7.2.0) [31]. The phylogenetic relationships were inferred by using the bayesian inference method and Hasegawa-Kishino-Yano nucleotide substitution model, where both country and year information were used to as a priors to run the analysis for 10 million states. BEAST suite (https://doi.org/10.1371/journal.pcbi.1003537) was used for Bayesian phylogenetics and FIGTREE was used to vizulized the tree (http://tree.bio.ed.ac.uk/software/figtree/).

## Results

### RT-PCR and nested PCR results

Out of 482 serum samples, 294 samples were positive for DENV and amplified 511 bp amplicon by consensus primers D1 and D2. Subsequently, 173 (58.8%) samples were serotyped as DENV-2 by nested PCR (named as A samples) while there was no amplification for 121 (41.2%) samples (named as B samples).

### Bioinformatics analysis

Thirty samples of those serotyped as DENV-2 by the serotype-specific primers (A samples) were sequenced to confirm the nested PCR results. The BLAST search for A samples sequences confirmed the nested PCR for serotype two and showed that the sequences were similar to DENV-2 isolated from Jazan in 2016 (accession number MK204365.1). A samples sequences also showed high similarity with published sequences of DENV-2 isolated mainly from India and Sudan (Table1).

**Table 1:** Accession numbers used to reconstruct the phylogenetic tree, ordered by the years. The (*) represents the sequences generated in this study

Accession	Groupe	Serotype	Country	City	Year
MG053122.1	A samples	DENV2	India	unknown	2010
JN935383.1	A samples	DENV2	India	Kerala	2010
JN935394.1	A samples	DENV2	India	Kerala	2010
KJ438865.1	A samples	DENV2	India	unknown	2012
KJ438871.1	A samples	DENV2	India	unknown	2012
MH645464.1	A samples	DENV2	India	Bangalore	2013
MH645454.1	A samples	DENV2	India	Chittoor	2013
KT180243.1	A samples	DENV2	India	unknown	2013
KT180256.1	A samples	DENV2	India	unknown	2014
MH048672.1	B samples	DENV2	Malaysia	unknown	2014
MH488959.1	B samples	DENV2	Malaysia	Johor Bahru	2015
MK513444.1	B samples	DENV2	Singapore	unknown	2015
MK629884.1	B samples	DENV2	South Korea	unknown	2015
MK204365.1	A samples	DENV2	Saudi Arabia	Jazan	2016
MF574722.1	A samples	DENV2	Sudan	Kassala	2016
MF441493.1	A samples	DENV2	India	unknown	2016
MG053122.1	A samples	DENV2	India	unknown	2016
MN018360.1	B samples	DENV2	China	Guangdong	2017
MN018361.1	B samples	DENV2	China	Guangdong	2017
MH110603.1	B samples	DENV2	China	Hangzhou	2017
MH827554.1	B samples	DENV2	China	unknown	2017
MK783189.1	B samples	DENV2	China	unknown	2017
MW512483.1	B samples	DENV2	Singapore	unknown	2018
OK048579.1*	B samples	DENV2	Saudi Arabia	Jazan	2019
MN923108.1	B samples	DENV2	China	unknown	2019
MN923110.1	B samples	DENV2	China	unknown	2019
MN923119.1	B samples	DENV2	China	unknown	2019
MN923121.1	B samples	DENV2	China	unknown	2019
MW512498.1	B samples	DENV2	Singapore	unknown	2019

A total of 121 samples that were first-round-PCR positive but D1-TS2 second-round-PCR negative (B samples) were sequenced and also subjected to BLAST search. C-prM region sequence generated during the present study was deposited in the GenBank database under accession number OK048579.1. B samples sequences showed high similarity with published sequences of DENV-2 isolated mainly from China, Malaysia, Singapore, and South_Korea (Table 1).

**Fig. 1:**
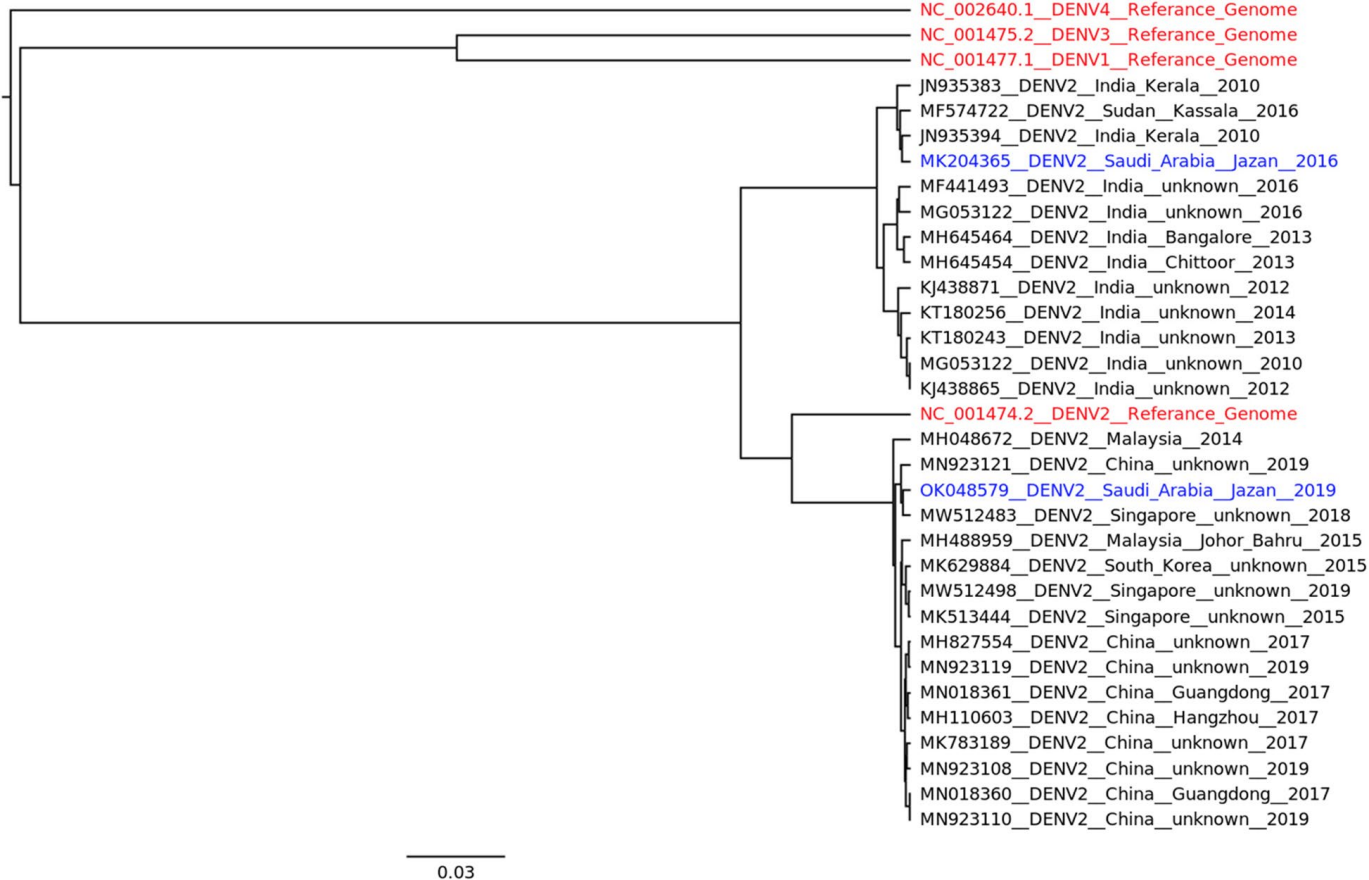
The evolutionary history was inferred by using the bayesian inference method. Red colour: reference samples; blue colour: samples from Jazan region and black colour: samples from different countries

Phylogenetic analysis using the Bayesian inference method (Fig. 1) revealed that DENV-2 viruses isolated from A samples and those isolated in 2016 were closely related to the Indian subcontinent lineages of the Cosmopolitan genotype of DENV-2 viruses. B samples, on the other hand, were closely related to Southeast Asian lineages.

### Differences in sequence between A and B samples

**Fig. 2:**
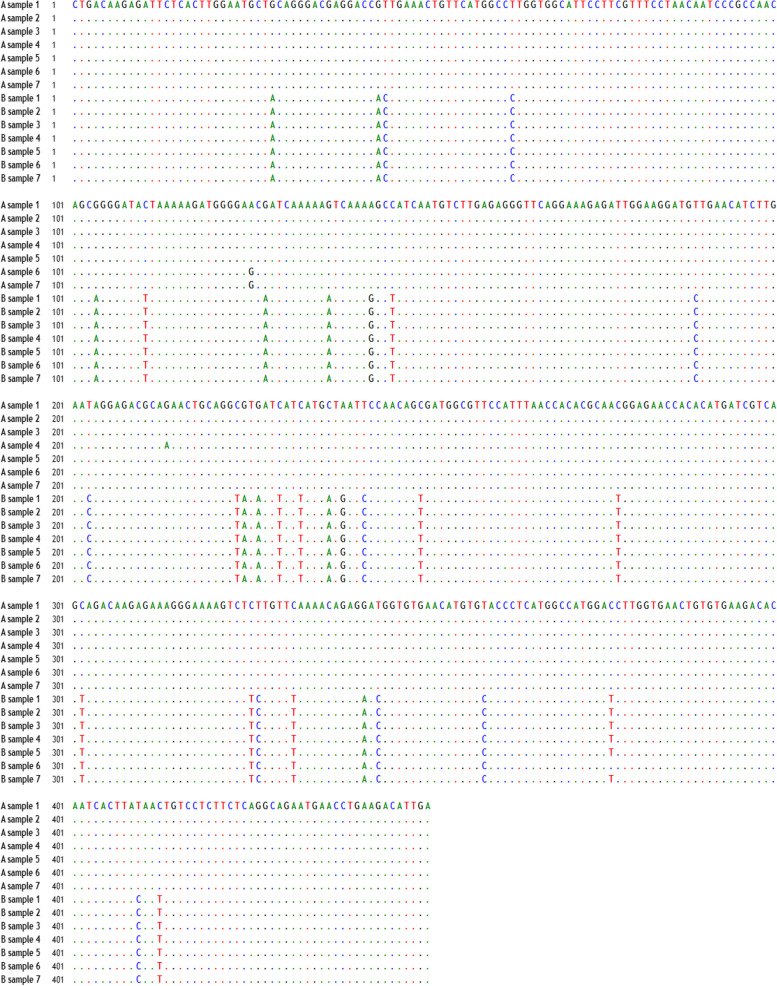
Alignment of DENV partial sequences of seven samples representing group A and seven samples representing group B. The alignment shows the differences in nucleotides between the two groups

BioEdit software was used to find the differences in sequences between A and B samples. The alignment (Fig. 2) and Table 2 show differences in 32 nucleotides in this part of sequences between A and B samples. similarity determined between all B samples sequenced were identical, except B sample 6 does not have the T substitution at position 386 seen in the other B sample sequences (Fig. 2).

**Table 2:** Nucleotide substitutions within C-prM region observed in the alignment between A and B sample groups

nucleotide position in alignment	A samples	B samples	nucleotide position in alignment	nucleotide A samples	nucleotide B samples
29	G	A	233	C	T
44	G	A	237	C	A
45	T	C	239	A	G
63	T	C	242	T	C
104	G	A	250	C	T
111	C	T	278	C	T
128	G	A	302	C	T
137	G	A	326	C	T
143	A	G	327	T	C
146	C	T	332	C	T
189	T	C	342	G	A
203	T	C	344	T	C
224	C	T	359	T	C
225	G	A	377	C	T
227	G	A	410	T	C
230	C	T	413	C	T

## Discussion

In Jazan region of southwestern Saudi Arabia, several outbreaks of dengue fever have occurred since 2005. A total of 4,619 dengue cases were reported between 2006 and 2020, with the highest number occurring in 2019 (1,654 cases), followed by 876 cases in 2020 [17, 22]. The topography and the subtropical nature of the region, along with its higher population density, have contributed to the incidence of those outbreaks. Recent studies have demonstrated that three dengue virus serotypes DENV-1, DENV-2 and DENV-3 are circulating in the region, with DENV-2 the most common serotype [17, 32, 33].

This study reports on the detection and sequence analysis of DENV-2 isolated from serum samples during the 2019 outbreak in Jazan region. Serotype-specific RT-PCR results yielded two groups of samples: A samples comprising DENV-2 isolates and B samples comprising DENV isolates that were negative for DENV serotyping. The Lanciotti protocol is widely used for diagnosis and surveillance of dengue [34, 35], and has been used by the SCDC in Jazan for the past decade. The present study took precautions to avoid laboratory errors and invalidate results. Furthermore, the utilised assay successfully serotyped 58.8% (173 of 294) of the samples as DENV-2. Therefore, it was hypothesised that group B samples detected during the 2019 outbreak were imported variant of DENV-2.

Subsequently, sequencing was performed to test the hypothesis and investigate the two groups' phylogenetic relationship. The sequence analysis results confirmed that A samples were DENV-2 belonging to the Cosmopolitan genotype circulating in the region since 2005 [18, 19]. Interestingly, the phylogenetic analysis for B samples revealed that these isolates were different from the Cosmopolitan genotype, with a total of 32 nucleotide substitutions (Table 2, Fig. 2). Therefore, these results support the hypothesis that the DENV-2 (B samples) isolated in Jazan region during the 2019 outbreak was imported genetic variant of DENV-2 that reported for the first time in the region.

It has been suggested that genetic variations in the viral genome of fast-evolving RNA virus populations such as DENV increase the likelihood of introducing mismatches into primer or probe binding regions and result in RT-PCR false-negative results [34]. Indeed, DENV variants that escape identification with commercially available type-specific monoclonal antibodies and RT-PCR using DENV type-specific primers have been reported in other countries [23, 35–37]. However, based on sequencing and bioinformatics analyses, this was not the case in this study.

The results showed that the sequence of the isolated DENV-2 imported variant was similar to the DENV-2 isolated in some Southeast Asian countries, including Malaysia, Singapore, the Republic of Korea and China. Hence, it can be postulated that workers might import this variant from those countries when entering the region. Conversely, the imported DENV-2 variant could help explain the dramatic increase in cases in 2019 and 2020, coinciding with the absence of any observed change in other factors associated with dengue transmission.

There is a scarcity of information about introduced DENV serotypes and genotypes in Saudi Arabia. Zaki et al. [18] showed that the Cosmopolitan genotype was the predominant DENV-2 genotype circulating during outbreaks in Jeddah between 1994 and 2006, and it was closely related to isolates from Australia, Thailand and Singapore. El-Kafrawy et al. [38] demonstrated that a different variant of DENV-2 Cosmopolitan genotype, with three to nine nucleotide substitutions compared to other previously reported DENV-2 strains, was responsible for the outbreak in Jeddah in 2014. Thus, the high number of imported variant DENV-2 isolates reported by this study is a major concern and requires continuous monitoring throughout the region.

Although the effect of genetic diversity between DENV serotypes and genotypes on clinical outcome is still controversial, the severity and endemicity of DENV have been linked to the imported variants in outbreaks in endemic areas [24, 39–42]. DENV-2 is the serotype commonly responsible for large epidemics in Asia and Latin America, with secondary DENV-2 infection associated with more severe disease [43–45]. The introduction of a new genetic variant was related to an increase in severe DENV cases in endemic areas [12, 46]. Unfortunately, determining the clinical severity associated with different genotypes of DENV-2 was not possible in the present study due to a lack of data. However, it is essential to monitor the spread of DENV serotypes, genotypes, and variants circulating in endemic areas to better understand the pathogenesis of DENV. Furthermore, the present study focused on the C-prM region sequence analysis. Indeed, previous studies have focused on the sequence analyses of viral individual genes or a subset of those genes, with the envelope (E) gene and C-prM region are the most frequently selected targets for DENV phylogenetic investigations and genotyping [34, 36, 47, 48]. However, a recent comparative analysis of viral mutations and evolution demonstrated significant differences when DENV whole-genome sequences were analyzed instead of individual genes [49]. Therefore, additional studies with whole DENV genome sequencing might provide critical information about the genetic relation between DENV isolates circulating in Jazan region and variants circulating in other regions.

## Conclusions

This study revealed that the imported genetic variant of DENV-2 was introduced to Jazan region during the 2019 outbreak, and it may have caused a dramatic increase in DENV cases in the region in 2019 and 2020. The isolated variant nucleotide identity showed 99.55% similarity with DENV-2 sequences reported from Singapore, Malaysia, and China. The emergence and co-circulation of new genetic variants of DENV serotypes can impact transmission dynamics, epidemic potential and disease severity. Therefore, the findings call for further investigations and large-scale continuous surveillance of DENV serotypes and genotypes circulating in Jazan region to better understand key drivers of dengue viral dynamics and epidemic patterns in southwestern Saudi Arabia.

## Data Availability

The data that support the findings of this study are available from the corresponding authors upon reasonable request. A representative sequence of the newly generated sequences was submitted to the GenBank database and received the accession number: OK048579.1.
